# (2,4-Dichloro­phen­yl)(diphenyl­phosphor­yl)methanol

**DOI:** 10.1107/S1600536809055548

**Published:** 2010-01-30

**Authors:** Wan-Yun Liu, Ping Huo, Tong-Lin Huang, Guang-Quan Mei

**Affiliations:** a Key Laboratory of Jiangxi University for Applied Chemistry and Chemical Biology, Yichun 336000, People’s Republic of China; b Jiangxi Science & Technology Research Center for Work Safety, Nanchang 330046, People’s Republic of China

## Abstract

In the title compound, C_19_H_15_Cl_2_O_2_P, the dihedral angle between the mean planes of the phenyl rings bonded to the P atom is 75.4 (1)°. In the crystal, mol­ecules are linked into chains running along the *a* axis by inter­molecular O—H⋯O hydrogen bonds. Mol­ecules are further connected into a three-dimensional array by weak C—H⋯O inter­actions.

## Related literature

For applications of the analogous compound (diphenyl­phosphino­yl)phenyl­methanol, see: Clark *et al.* (2002 ▶). For related structures, see: Liu *et al.* (2007 ▶); Liu & Huo (2008 ▶).
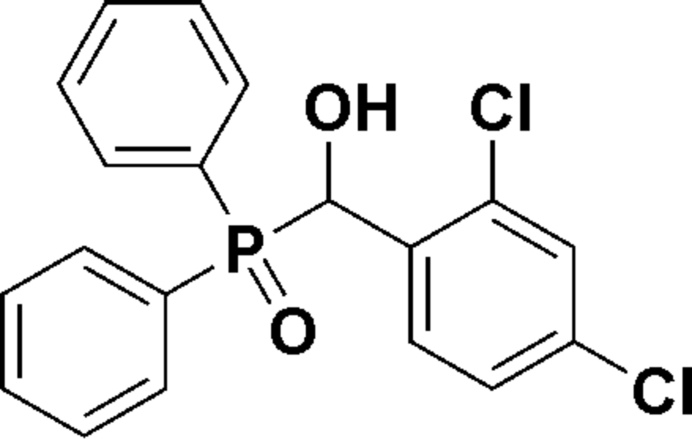


## Experimental

### 

#### Crystal data

C_19_H_15_Cl_2_O_2_P
*M* *_r_* = 377.18
Monoclinic, 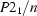

*a* = 8.8157 (18) Å
*b* = 11.334 (2) Å
*c* = 19.262 (4) Å
β = 102.41 (3)°
*V* = 1879.6 (7) Å^3^
*Z* = 4
Mo *K*α radiation
μ = 0.44 mm^−1^
*T* = 293 K
0.30 × 0.23 × 0.12 mm

#### Data collection

Bruker SMART APEX area-detector diffractometer
Absorption correction: multi-scan (*SADABS*; Bruker, 2001 ▶) *T*_min_ = 0.880, *T*_max_ = 0.949
15866 measured reflections
3680 independent reflections
2783 reflections with *I* > 2σ(*I*)
*R*_int_ = 0.025

#### Refinement

*R*[*F* ^2^ > 2σ(*F* ^2^)] = 0.049
*wR*(*F* ^2^) = 0.156
*S* = 1.11
3680 reflections
217 parameters
H-atom parameters constrained
Δρ_max_ = 0.43 e Å^−3^
Δρ_min_ = −0.44 e Å^−3^

### 

Data collection: *SMART* (Bruker, 2001 ▶); cell refinement: *SAINT* (Bruker, 2001 ▶); data reduction: *SAINT*; program(s) used to solve structure: *SHELXS97* (Sheldrick, 2008 ▶); program(s) used to refine structure: *SHELXL97* (Sheldrick, 2008 ▶); molecular graphics: *ORTEP-3 for Windows* (Farrugia, 1997 ▶); software used to prepare material for publication: *SHELXL97*.

## Supplementary Material

Crystal structure: contains datablocks I, global. DOI: 10.1107/S1600536809055548/pv2247sup1.cif

Structure factors: contains datablocks I. DOI: 10.1107/S1600536809055548/pv2247Isup2.hkl

Additional supplementary materials:  crystallographic information; 3D view; checkCIF report

## Figures and Tables

**Table 1:** Hydrogen-bond geometry (Å, °)

*D*—H⋯*A*	*D*—H	H⋯*A*	*D*⋯*A*	*D*—H⋯*A*
O2—H2*A*⋯O1^i^	0.82	1.79	2.576 (2)	161
C10—H10*A*⋯O2^ii^	0.93	2.64	3.348 (3)	134
C1—H1*A*⋯O1^i^	0.98	2.69	3.075 (3)	104

Symmetry codes: (i) 
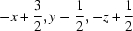
; (ii) 
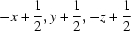
.

**Fig. 1.**
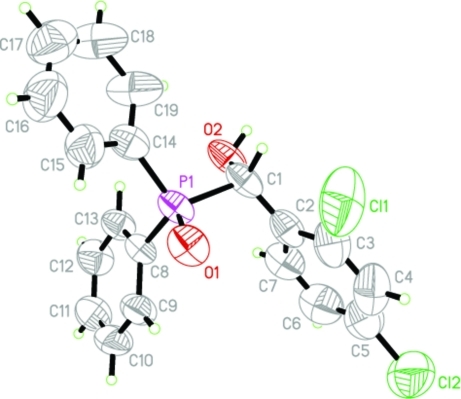
The molecular structure of (I), showing 50% probability displacement ellipsoids (arbitrary spheres for H atoms).

## supplementary crystallographic information

### Comment

The title compound, (I), is an analog of (diphenylphosphinoyl)phenylmethanol, which was employed as a ligand in the rhodium-catalyzed hydroformylation of alkenes, with good conversions and regioselectivities (Clark *et al.*, 2002).

The molecular structure of (I) is shown in Fig. 1. Bond lengths and angles in (I) are in agreement with those reported for similar compounds (Liu *et al.*, 2007; Liu *et al.*, 2008). The dihedral angle between the mean-planes of the phenyl rings (C8—C13) and (C14—C19) bonded to P-atoms is 75.4 (1)°. A strong O—H···O hydrogen bond involving the hydroxyl group link the molecules into a chain running along the *a* axis (Table 1). Molecules are further connected into a three-dimensional array by non-classical and rather weak C—H···O intermolecular hydrogen-bonding interactions.

### Experimental

To a solution of 2, 4-dichlorobenzaldehyde (0.35 g, 2.0 mmol) and diphenylphosphine oxide (0.40 g, 2.0 mmol) in tetrahydrofuran (10 ml) at 273 K was added dropwise triethylamine (0.03 ml, 2 mmol). The cooling bath was removed and the mixture warmed to ambient temperature for 2 h. The solvent was concentrated under vacuum and the crude product was purified by recrystallization in methanol to give the title compound as a white solid in 80% yield. Single crystals of (I) were obtained by slow evaporation of a methanol solution.

### Refinement

All H atoms were placed in geometrically idealized positions and constrained to ride on their parent atoms, with C—H = 0.93 Å (aromatic), 0.98 Å (methine), O—H = 0.82 Å, and *U*_iso_(H) = 1.2*U*_eq_(*c*) and 1.5*U*_eq_(O).

### Crystal data

**Table:**

C_19_H_15_Cl_2_O_2_P	*F*(000) = 776
*M**_r_* = 377.18	*D*_x_ = 1.333 Mg m^−^^3^
Monoclinic, *P*2_1_/*n*	Mo *K*α radiation, λ = 0.71073 Å
Hall symbol: -P 2yn	Cell parameters from 1526 reflections
*a* = 8.8157 (18) Å	θ = 3.3–27.5°
*b* = 11.334 (2) Å	µ = 0.44 mm^−^^1^
*c* = 19.262 (4) Å	*T* = 293 K
β = 102.41 (3)°	Plate, colorless
*V* = 1879.6 (7) Å^3^	0.30 × 0.23 × 0.12 mm
*Z* = 4	

### Data collection

**Table:**

Bruker APEX area-detector diffractometer	3680 independent reflections
Radiation source: fine-focus sealed tube	2783 reflections with *I* > 2σ(*I*)
graphite	*R*_int_ = 0.025
φ and ω scans	θ_max_ = 26.0°, θ_min_ = 3.3°
Absorption correction: multi-scan (*SADABS*; Bruker, 2001)	*h* = −10→10
*T*_min_ = 0.880, *T*_max_ = 0.949	*k* = −13→13
15866 measured reflections	*l* = −23→23

### Refinement

**Table:**

Refinement on *F*^2^	Primary atom site location: structure-invariant direct methods
Least-squares matrix: full	Secondary atom site location: difference Fourier map
*R*[*F*^2^ > 2σ(*F*^2^)] = 0.049	Hydrogen site location: inferred from neighbouring sites
*wR*(*F*^2^) = 0.156	H-atom parameters constrained
*S* = 1.11	*w* = 1/[σ^2^(*F*_o_^2^) + (0.0771*P*)^2^ + 0.5515*P*] where *P* = (*F*_o_^2^ + 2*F*_c_^2^)/3
3680 reflections	(Δ/σ)_max_ < 0.001
217 parameters	Δρ_max_ = 0.43 e Å^−^^3^
0 restraints	Δρ_min_ = −0.44 e Å^−^^3^

### Special details

**Table:**

Geometry. All e.s.d.'s (except the e.s.d. in the dihedral angle between two l.s. planes) are estimated using the full covariance matrix. The cell e.s.d.'s are taken into account individually in the estimation of e.s.d.'s in distances, angles and torsion angles; correlations between e.s.d.'s in cell parameters are only used when they are defined by crystal symmetry. An approximate (isotropic) treatment of cell e.s.d.'s is used for estimating e.s.d.'s involving l.s. planes.
Refinement. Refinement of *F*^2^ against ALL reflections. The weighted *R*-factor *wR* and goodness of fit *S* are based on *F*^2^, conventional *R*-factors *R* are based on *F*, with *F* set to zero for negative *F*^2^. The threshold expression of *F*^2^ > σ(*F*^2^) is used only for calculating *R*-factors(gt) *etc*. and is not relevant to the choice of reflections for refinement. *R*-factors based on *F*^2^ are statistically about twice as large as those based on *F*, and *R*- factors based on ALL data will be even larger.

### Fractional atomic coordinates and isotropic or equivalent isotropic displacement parameters (Å^2^)

**Table:**

	*x*	*y*	*z*	*U*_iso_*/*U*_eq_	
P1	0.63438 (6)	0.94073 (5)	0.21088 (4)	0.0543 (2)	
Cl1	0.99338 (10)	0.89244 (14)	0.36475 (6)	0.1424 (5)	
Cl2	0.66962 (19)	0.89703 (11)	0.57032 (6)	0.1442 (5)	
C1	0.6904 (2)	0.80652 (19)	0.26412 (15)	0.0612 (6)	
H1A	0.7951	0.7831	0.2601	0.073*	
C2	0.6902 (3)	0.8293 (2)	0.34053 (15)	0.0661 (6)	
C3	0.8186 (4)	0.8676 (3)	0.38994 (19)	0.0881 (9)	
C4	0.8124 (5)	0.8865 (3)	0.4608 (2)	0.1068 (12)	
H4A	0.9007	0.9108	0.4934	0.128*	
C5	0.6769 (5)	0.8695 (3)	0.48191 (19)	0.0970 (10)	
C6	0.5478 (5)	0.8317 (3)	0.4349 (2)	0.1001 (11)	
H6A	0.4555	0.8194	0.4499	0.120*	
C7	0.5547 (3)	0.8117 (3)	0.36529 (18)	0.0799 (8)	
H7A	0.4660	0.7856	0.3337	0.096*	
C8	0.4292 (2)	0.95822 (18)	0.20306 (12)	0.0524 (5)	
C9	0.3801 (3)	1.0514 (2)	0.24046 (15)	0.0701 (7)	
H9A	0.4526	1.1014	0.2679	0.084*	
C10	0.2238 (3)	1.0687 (3)	0.23653 (17)	0.0839 (9)	
H10A	0.1909	1.1313	0.2608	0.101*	
C11	0.1173 (3)	0.9947 (3)	0.19732 (17)	0.0839 (9)	
H11A	0.0122	1.0067	0.1955	0.101*	
C12	0.1633 (3)	0.9022 (3)	0.16022 (17)	0.0781 (8)	
H12A	0.0896	0.8523	0.1334	0.094*	
C13	0.3199 (3)	0.8840 (2)	0.16314 (14)	0.0618 (6)	
H13A	0.3516	0.8217	0.1382	0.074*	
C14	0.6700 (3)	0.9144 (2)	0.12355 (16)	0.0662 (6)	
C15	0.6984 (4)	1.0121 (3)	0.08557 (17)	0.0890 (9)	
H15A	0.6956	1.0868	0.1053	0.107*	
C16	0.7307 (5)	1.0012 (4)	0.0193 (2)	0.1152 (12)	
H16A	0.7501	1.0682	−0.0053	0.138*	
C17	0.7344 (5)	0.8922 (5)	−0.0107 (2)	0.1150 (13)	
H17A	0.7546	0.8846	−0.0559	0.138*	
C18	0.7083 (5)	0.7958 (4)	0.0261 (3)	0.1295 (16)	
H18A	0.7117	0.7216	0.0059	0.155*	
C19	0.6763 (5)	0.8045 (3)	0.0935 (2)	0.1076 (12)	
H19A	0.6594	0.7369	0.1181	0.129*	
O1	0.7200 (2)	1.04574 (14)	0.24533 (11)	0.0708 (5)	
O2	0.58404 (18)	0.71745 (13)	0.23355 (11)	0.0705 (5)	
H2A	0.6292	0.6539	0.2359	0.106*	

### Atomic displacement parameters (Å^2^)

**Table:**

	*U*^11^	*U*^22^	*U*^33^	*U*^12^	*U*^13^	*U*^23^
P1	0.0408 (3)	0.0380 (3)	0.0837 (4)	−0.0029 (2)	0.0128 (3)	0.0001 (3)
Cl1	0.0633 (5)	0.2302 (15)	0.1210 (8)	−0.0437 (7)	−0.0081 (5)	0.0291 (8)
Cl2	0.2145 (15)	0.1219 (9)	0.1001 (7)	0.0202 (9)	0.0428 (8)	0.0175 (6)
C1	0.0386 (11)	0.0413 (11)	0.1017 (19)	0.0011 (8)	0.0105 (11)	0.0028 (11)
C2	0.0552 (13)	0.0450 (12)	0.0951 (19)	0.0006 (10)	0.0097 (12)	0.0127 (12)
C3	0.0698 (18)	0.090 (2)	0.098 (2)	−0.0106 (15)	0.0030 (15)	0.0224 (18)
C4	0.111 (3)	0.103 (3)	0.092 (2)	−0.011 (2)	−0.009 (2)	0.021 (2)
C5	0.125 (3)	0.073 (2)	0.094 (2)	0.006 (2)	0.025 (2)	0.0176 (17)
C6	0.105 (3)	0.084 (2)	0.122 (3)	0.0023 (19)	0.046 (2)	0.014 (2)
C7	0.0708 (17)	0.0644 (16)	0.108 (2)	−0.0025 (13)	0.0265 (15)	0.0054 (15)
C8	0.0456 (11)	0.0439 (11)	0.0677 (13)	0.0078 (8)	0.0121 (9)	0.0042 (10)
C9	0.0613 (15)	0.0642 (15)	0.0818 (17)	0.0153 (12)	0.0088 (12)	−0.0097 (13)
C10	0.0691 (18)	0.094 (2)	0.0890 (19)	0.0313 (15)	0.0182 (15)	−0.0146 (16)
C11	0.0480 (14)	0.109 (2)	0.095 (2)	0.0237 (15)	0.0167 (13)	−0.0002 (18)
C12	0.0464 (14)	0.0899 (19)	0.094 (2)	−0.0003 (12)	0.0063 (13)	−0.0071 (16)
C13	0.0474 (12)	0.0583 (13)	0.0789 (16)	0.0034 (10)	0.0118 (11)	−0.0054 (12)
C14	0.0440 (12)	0.0645 (15)	0.0914 (18)	−0.0019 (10)	0.0178 (11)	−0.0042 (13)
C15	0.106 (2)	0.078 (2)	0.082 (2)	0.0025 (17)	0.0163 (17)	0.0079 (16)
C16	0.143 (3)	0.115 (3)	0.091 (2)	−0.003 (3)	0.031 (2)	0.016 (2)
C17	0.105 (3)	0.146 (4)	0.101 (3)	−0.002 (3)	0.038 (2)	−0.013 (3)
C18	0.150 (4)	0.108 (3)	0.155 (4)	−0.013 (3)	0.088 (3)	−0.043 (3)
C19	0.127 (3)	0.079 (2)	0.140 (3)	−0.0133 (19)	0.081 (3)	−0.023 (2)
O1	0.0637 (10)	0.0464 (9)	0.0993 (13)	−0.0165 (7)	0.0111 (9)	−0.0006 (8)
O2	0.0504 (9)	0.0381 (8)	0.1200 (15)	0.0003 (6)	0.0116 (9)	0.0005 (9)

### Geometric parameters (Å, °)

**Table:**

P1—O1	1.4872 (17)	C9—H9A	0.9300
P1—C8	1.794 (2)	C10—C11	1.360 (4)
P1—C14	1.801 (3)	C10—H10A	0.9300
P1—C1	1.842 (2)	C11—C12	1.378 (4)
Cl1—C3	1.735 (3)	C11—H11A	0.9300
Cl2—C5	1.746 (4)	C12—C13	1.385 (3)
C1—O2	1.417 (3)	C12—H12A	0.9300
C1—C2	1.495 (4)	C13—H13A	0.9300
C1—H1A	0.9800	C14—C15	1.379 (4)
C2—C3	1.383 (4)	C14—C19	1.380 (4)
C2—C7	1.393 (4)	C15—C16	1.372 (5)
C3—C4	1.394 (5)	C15—H15A	0.9300
C4—C5	1.356 (5)	C16—C17	1.368 (6)
C4—H4A	0.9300	C16—H16A	0.9300
C5—C6	1.363 (5)	C17—C18	1.349 (6)
C6—C7	1.374 (5)	C17—H17A	0.9300
C6—H6A	0.9300	C18—C19	1.389 (5)
C7—H7A	0.9300	C18—H18A	0.9300
C8—C13	1.382 (3)	C19—H19A	0.9300
C8—C9	1.398 (3)	O2—H2A	0.8200
C9—C10	1.378 (4)		
			
O1—P1—C8	110.82 (11)	C10—C9—H9A	120.1
O1—P1—C14	112.05 (11)	C8—C9—H9A	120.1
C8—P1—C14	108.36 (11)	C11—C10—C9	120.3 (3)
O1—P1—C1	111.28 (11)	C11—C10—H10A	119.8
C8—P1—C1	106.43 (10)	C9—C10—H10A	119.8
C14—P1—C1	107.67 (12)	C10—C11—C12	120.8 (2)
O2—C1—C2	113.1 (2)	C10—C11—H11A	119.6
O2—C1—P1	106.44 (16)	C12—C11—H11A	119.6
C2—C1—P1	110.43 (16)	C11—C12—C13	119.6 (3)
O2—C1—H1A	108.9	C11—C12—H12A	120.2
C2—C1—H1A	108.9	C13—C12—H12A	120.2
P1—C1—H1A	108.9	C8—C13—C12	120.1 (2)
C3—C2—C7	116.4 (3)	C8—C13—H13A	120.0
C3—C2—C1	123.9 (2)	C12—C13—H13A	120.0
C7—C2—C1	119.7 (2)	C15—C14—C19	118.3 (3)
C2—C3—C4	121.5 (3)	C15—C14—P1	116.8 (2)
C2—C3—Cl1	120.2 (3)	C19—C14—P1	124.9 (2)
C4—C3—Cl1	118.2 (3)	C16—C15—C14	121.2 (3)
C5—C4—C3	119.7 (3)	C16—C15—H15A	119.4
C5—C4—H4A	120.2	C14—C15—H15A	119.4
C3—C4—H4A	120.2	C17—C16—C15	120.2 (4)
C4—C5—C6	120.6 (4)	C17—C16—H16A	119.9
C4—C5—Cl2	119.2 (3)	C15—C16—H16A	119.9
C6—C5—Cl2	120.2 (3)	C18—C17—C16	119.1 (4)
C5—C6—C7	119.6 (3)	C18—C17—H17A	120.4
C5—C6—H6A	120.2	C16—C17—H17A	120.4
C7—C6—H6A	120.2	C17—C18—C19	121.7 (4)
C6—C7—C2	122.1 (3)	C17—C18—H18A	119.1
C6—C7—H7A	118.9	C19—C18—H18A	119.1
C2—C7—H7A	118.9	C14—C19—C18	119.4 (4)
C13—C8—C9	119.4 (2)	C14—C19—H19A	120.3
C13—C8—P1	123.32 (17)	C18—C19—H19A	120.3
C9—C8—P1	117.30 (19)	C1—O2—H2A	109.5
C10—C9—C8	119.8 (3)		
			
O1—P1—C1—O2	170.00 (15)	O1—P1—C8—C9	−11.8 (2)
C8—P1—C1—O2	49.18 (19)	C14—P1—C8—C9	−135.1 (2)
C14—P1—C1—O2	−66.84 (18)	C1—P1—C8—C9	109.4 (2)
O1—P1—C1—C2	46.92 (18)	C13—C8—C9—C10	−0.6 (4)
C8—P1—C1—C2	−73.91 (18)	P1—C8—C9—C10	−179.8 (2)
C14—P1—C1—C2	170.08 (15)	C8—C9—C10—C11	0.9 (5)
O2—C1—C2—C3	150.1 (2)	C9—C10—C11—C12	−0.8 (5)
P1—C1—C2—C3	−90.8 (3)	C10—C11—C12—C13	0.3 (5)
O2—C1—C2—C7	−29.9 (3)	C9—C8—C13—C12	0.2 (4)
P1—C1—C2—C7	89.2 (2)	P1—C8—C13—C12	179.3 (2)
C7—C2—C3—C4	0.5 (4)	C11—C12—C13—C8	0.0 (4)
C1—C2—C3—C4	−179.5 (3)	O1—P1—C14—C15	−31.3 (3)
C7—C2—C3—Cl1	−179.9 (2)	C8—P1—C14—C15	91.3 (2)
C1—C2—C3—Cl1	0.1 (4)	C1—P1—C14—C15	−153.9 (2)
C2—C3—C4—C5	−1.3 (5)	O1—P1—C14—C19	146.3 (3)
Cl1—C3—C4—C5	179.1 (3)	C8—P1—C14—C19	−91.1 (3)
C3—C4—C5—C6	1.4 (6)	C1—P1—C14—C19	23.6 (3)
C3—C4—C5—Cl2	−178.5 (3)	C19—C14—C15—C16	0.6 (5)
C4—C5—C6—C7	−0.6 (6)	P1—C14—C15—C16	178.3 (3)
Cl2—C5—C6—C7	179.2 (3)	C14—C15—C16—C17	0.4 (6)
C5—C6—C7—C2	−0.2 (5)	C15—C16—C17—C18	−1.0 (7)
C3—C2—C7—C6	0.3 (4)	C16—C17—C18—C19	0.6 (7)
C1—C2—C7—C6	−179.7 (3)	C15—C14—C19—C18	−1.0 (6)
O1—P1—C8—C13	169.1 (2)	P1—C14—C19—C18	−178.6 (3)
C14—P1—C8—C13	45.8 (2)	C17—C18—C19—C14	0.4 (7)
C1—P1—C8—C13	−69.8 (2)		

### Hydrogen-bond geometry (Å, °)

**Table:**

*D*—H···*A*	*D*—H	H···*A*	*D*···*A*	*D*—H···*A*
O2—H2A···O1^i^	0.82	1.79	2.576 (2)	161
C10—H10A···O2^ii^	0.93	2.64	3.348 (3)	134
C1—H1A···O1^i^	0.98	2.69	3.075 (3)	104

Symmetry codes: (i) −*x*+3/2, *y*−1/2, −*z*+1/2; (ii) −*x*+1/2, *y*+1/2, −*z*+1/2.
